# Patterns of medication use and adherence to medications among residents in the elderly homes

**DOI:** 10.12669/pjms.36.4.1923

**Published:** 2020

**Authors:** Magda Algameel

**Affiliations:** Dr. Magda Algameel, Nursing Department, College of Applied Medical Science, Prince Sattam Bin Abdulaziz University, KSA, Wadi Al-Dawasser 11991, Saudi Arabia

**Keywords:** Institutionalized elders, Clinical pharmacist role, Medication regimens, Gerontological nurse role, Patient education

## Abstract

**Objective:**

To evaluate health care related to medication regimens among institutionalized elders in Damanhour, Beheira Governate, Egypt.

**Methods:**

A prospective, multi-centered, observational study was conducted in the two elderly residential homes in Damanhour between March and May 2017. A questionnaire was developed and validated to test for elderly socio-economic, chronic diseases, current therapy adherence, vaccination history and patient education. Descriptive and quantitative analysis were performed.

**Results:**

sixty-three elderly residents were included in the study. The sample showed broad socioeconomic variability posing a true reflection of Egyptian population. 63.5% had no hearing problems, 31.7% had proper vision and 57% could move with no help. More than three quarters had chronic diseases of which 58.7% were previously hospitalized. The most prevalent diseases were hypertension, diabetes and arthritis 46%, 41.3%, 26.9% respectively. Only 7.9% and 4.7% showed chronic liver and kidney diseases, respectively and less than 10% suffered from respiratory related diseases. No alcohol drinker, 25.3% were smokers and 58.7% drank caffeine. Only 25.3% of residents showed full adherence to their medication pattern. Approximately 80% of residents never received proper patient education. Forty-three residents did not know the indication of their medications and 92% ignored its side effects.

**Conclusion:**

Absence of proper medical care exposure for the elderly residents was reflected in their low medication adherence, adverse side effects and hospitalization. We suggest extension of the national medical insurance system to include larger number of elderly population. To monitor the care given concerning medication, a daily resident gerontological nurse needs to be assigned, visits by clinical pharmacists weekly or bi-weekly from the nearby governmental hospital can improve improper medication.

## INTRODUCTION

For the past decade, people have been living longer and the population is aging rapidly.^1^ In Egypt, elderly population is expected to increase from 6.1% in 2006 to 15% by 2051. The number of elderly homes was reported to be about 108 by 2007.^2^ Elderly population usually has an increased demand on healthcare services, where they experience multiple diseases and use more medication than younger age groups.^3^ Thus the current challenge for healthcare professionals has shifted from increasing people’s life span to achieving a better quality of life for them.^1^ Recent studies reveal that elderly population living in residential/nursing homes consume up to four times more medications than those who do not reside in nursing homes.^4^ Pharmacotherapy management in elderly is challenging due to presence of comorbidities, age-related alterations in drugs pharmacokinetics, pharmacodynamics and polypharmacy which may result in drug-drug &/or drug disease interactions. This requires special care and involvement from the whole healthcare team including physicians, pharmacists and nurses.^5^

The pharmacist and nurse roles are mostly centered to monitor and manage medication regimens care through providing enough education and awareness to such population about their current diseases, therapy goals, therapy management and help to follow up the elderly population health progress. In absence of proper medication related care in such homes, elderly residents are more prone to using medications for inappropriate indications, taking wrong doses, suffering from medication non-adherence issues, more side effects and possibly more drug interactions.^6^,^7^

The manuscript aims to identify reflections on Improper administration of medications among residents in the elderly homes.

## METHODS

This prospective, multi-centered, observational study was approved and supported by Institutional Review Board of Faculty of Nursing, Damnhour University (Ref.No. 2017/02/44399). Official letters were issued from the Faculty of Nursing, Damnhour University to the directors of the elderly homes for administrative approvals. The study covered the only two elderly residential homes in Damanhour, Beheira Governate were involved for the study. The only two homes, one governmental with Forty residents and the other is private with Thirty residents.

Residents were included in the study upon fulfilling the following inclusion criteria:

- Has 60 years or more.
- Has no cognitive impairment.
- Is willing to participate in the study.

### Tools construction and validation

A questionnaire was developed by the researcher to test for socio-economic status of the elderly population, their chronic diseases, medications taking & medication adherence and medical knowledge. The questionnaire was self-administered by the elderly population through an interview with the researcher.

The questionnaire and structured interview was tested for the content validity by three Gerontological Nursing & Pharmacy Practice referees in Damanhour and Alexandria Universities. Validation was further confirmed by a pilot study that was carried out on a sample of seven elders selected randomly from “Dar El- Hanaa”, in Alexandria to evaluate the developed tools for their feasibility and applicability. In addition to the questionnaire the researcher used a Mini- mental State Examination (MMSE) developed by Folstein M in 1975 and translated to the Egyptian Arabic language by Elokl in 2002.^8^,^9^ The researcher used it to assess the elderly cognitive function, and thus select cognitively intact elders.

### Data collection

Data were collected from first March 2017 to the end of May 2017.

### Data analysis and statistics

After descriptive and quantitative data were collected, coded and analyzed. The statistical package for social sciences (SPSS 20.0) was utilized for data analysis and tabulation. Statistically significant at P ≤ 0.05. Data were coded.

## RESULTS

Out of the seventy elderly residents, sixty-three residents fulfilled the criteria and were included in the study. Thirty-three from “Al-Mogamae” and thirty from “Dar El-Saada” elderly home. The residents’ sample that fulfilled the criteria showed a broad socioeconomic variability thus they represent a true sample of the Egyptian elder society with ages ranging from 60 to greater than 85 year and number of both gender 33.3% males, 66.6% females. Their educational levels ranged from illiteracy till university professional degrees and they had variable monthly income and health habits. All residents did not drink alcohol, 25.3% were smokers and 58.7% reported drinking caffeine (Table-I).

**Table-I:** Distribution of the residential home elders according to Socio-demographic data (n = 63).

Variable	Category	Frequency	Percentage (%)
***Home name***			
	Elmogamaa	33	52.3
	El sadaa	30	47.6
***Age***			
	60-75	53	84.1
	75-85	8	12.6
	More than 85	2	3.1
***Gender***			
	Female	42	66.6
	Male	21	33.3
***Education***			
	Illiterate	34	53.9
	Read & write	2	3.1
	Primary	11	17.4
	Preparatory	3	4.7
	Secondary	9	14.3
	University	4	6.3
***Marital status***			
	Single	6	9.5
	Married	6	9.5
	Divorced	12	19.1
	Widow	39	61.9
***Occupation***			
	Prof work	7	11.1
	House wife	25	39.7
	Worker	12	19.1
	Commercial	5	7.9
	Officer	13	20.6
	Other	1	1.6
***Income***			
	Less than 300	1	1.6
	300 – 400	6	9.5
	400 – 500	27	42.9
	More than 500	29	46.03
***Source of income***			
	Pension	57	90.5
	Sons	6	9.5
	Relative	10	15.9
	Still working	1	1.6
	Other	2	3.1
***Health Habits & Risk behaviors***			
	Smokers	16	25.3
	Alcohol intake	0	0.0
	Caffeine intake	37	58.7

Table-II illustrates the chronic diseases and physical status of our residents where 63.5% had no hearing problems, 31.7% had proper vision, 57% could move by themselves and required no help. Looking at the health of our residents to determine the necessity of health care management for such population, We found that 82.5% of the elderly resident’s sample had chronic diseases. The most prevalent diseases were hypertension (46.05%), diabetes (41.3%) and arthritis (26.9.2%). Only 7.9% and 4.7% showed chronic liver and kidney diseases, respectively. Residents with respiratory related diseases (Asthma and COPD) were 9.5% of the residents. Regarding previous hospitalization 59% of the residents studied were hospitalized at least once before (Table-II).

**Table-II:** Distribution of the residential home elders according to Chronic diseases, physical status and number of hospital admissions (n = 63).

Variable	Category	Frequency	Percentage (%)
	Hypertension	29	46.03
	Diabetes	26	41.3
Residents with Chronic diseases	Arthritis	17	26.9
	Respiratory disorders	6	9.5
	Incontinence	7	11.1
	Liver diseases	5	7.9
	Kidney diseases	5	7.9
	Parkinson’s disease	3	4.8
Hearing quality			
	Good	40	63.5
	Difficult	19	30.2
	Use hearing aids	4	6.3
Vision quality			
	Good	20	31.7
	Difficult	23	36.5
	Use eye glass	20	31.7
Difficulties in using Hands			
	Arthritic	8	12.7
	Tremors	5	7.9
	Paralysis	0	0.0
Mobility of residents			
	Able to move by himself	36	27.1
	Needs assistance	18	28.6
	Uses walker or other tools	9	14.3
Number of hospital admissions			
	Never	26	41.3
	Once	21	33.3
	Twice	8	12.7
	More	8	12.7

Testing the healthcare services provided to the residents, it is worth mentioning that fifty out of the sixty-three residents did not receive proper education about their disease condition, medication use, expected therapy goals and the possible side effects. Those who received patient education (n=13) stated that they got that through doctors (7.9%), nurses (9.5%) and pharmacists (3.1%) (Table-III).

**Table-III:** Distribution of the residential home elders according to health education, medication knowledge, adherence and management of side effects (n = 63).

Variable	Category	Frequency	Percentage (%)
Health Education from			
	Nurses	6	9.5
	Doctors	5	7.9
	Pharmacists	2	3.1
Residents who managed to answer therapy Knowledge questions related to			
	Drug name and dose	21	33.3
	Dose time and interval	60	95.2
	Medication indication	36	57.1
	Possible side effects	5	7.9
Residents Medication adherence pattern			
	Fully adherent	16	25.3
	Somewhat adherent	39	62.0
	Not adherent	8	12.7
Side effects			
	Dizziness	20	31.7
	Nausea	12	19.4
	Coma	1	1.6
Management			
	Continue medication	3	4.8
	Decrease dose	10	15.9
	Increase dosing interval	5	7.9
	Consult a friends	2	3.2
	Consult pharmacist	3	4.8
	Consult physician	7	11.1
	Stop medication	6	9.5

* More than one response was given.

Testing the current medication knowledge of the elderly population sample, found that 66.7% did not know the drugs’ names or doses, 43% did not know the indication for the medication. In addition, the majority do not know the possible side effects of the medications they are using, however, thirty-four of them believe they suffer from side effects of their current therapy. The side effects reported were mostly dizziness (n=20) and nausea (n=12) as per the residents descriptions. Residents reacted to adverse drug events through different approaches where ten residents mentioned that they would decrease the dose or the frequency of the medications by themselves, seven persons would consult the physician, three would consult the pharmacist, three would continue taking their medications with no changes, six would stop the medications and two clients would consult his friends who had previous experiences.

Testing patient compliance in the elderly population sample, 25.3% were found to be adherent to their prescribed medications whereas 62% were (sometimes) trying to be compliant and about 12.7% were non-compliant (neglect their treatment). The two main reasons behind partial non-compliance were reported to be (a) reasons related to the medication: as medication size, packaging, price and availability (b) reasons related to the patient: as difficulty in remembering medication times, difficulty in purchasing the medications and difficulty in self-administering the medication (Table-IV).

**Table-IV:** Distribution of the residential home elders according to challenges that affects their adherence to medication (n = 63).

Variable	Category	Frequency	Percentage (%)
Challenges related to medication	Expensive	26	41.3
	Difficult to open	2	3.2
	Unavailability in the market	15	23.8
Challenges related to patient	Forgetting to take medication	39	61.9
	Cannot afford to buy	16	25.4
	In capable of Self-administering drugs	12	19.04
	Other	1	1.6
Patient behavior after first signs of improvement	Continue taking the medicine	3	4.8
	Stop taking the medicine	25	39.7
	decrease the frequency	15	23.3
	decreases the dose	7	11.1
	consult with healthcare provider	16	25.4
	Consult a friend	1	1.6

In fact, due to lack of proper patient education, most of residents did not realize the consequences of non-adherence. Asked about the reaction to question, when the patients accidentally forget to take medicines 61.9% said that they will take whenever they remember, 39.7% said that they stop medication once they feel the improvement in their health., 25.4% said they will consult the doctor before stopping the medication while, 23.8% said that they would decrease the dosage by themselves without proper advice, one person said that a friend would be consulted and three said that will continue taking their medications as prescribed (Table-IV).

The consumption of most common drugs by the inmates is as follows 35.2% use Anti-hypertension drugs (Atenolol, Reserpin), 30% Hypoglycemic (Metaformin, Insulin, Glimepiride), 12.7% Vitamins supplements (B,E), 22.1% over the counter medications used were non-steroidal anti-inflammatory drugs NSAID (Ibuprofen, Acetaminophen and drugs for treatment of cold and congestion.

## DISCUSSION

Patient medication compliance is defined as the extent to which a patient’s behavior matches the prescriber’s advice. Whereas, adherence indicates the collaboration of patient and physician to improve the patient’s health through integrating expert medical opinion with the patient’s lifestyle, values and preferences.^10^,^11^ In absence of proper patient education, compliance and adherence to medication becomes challenging for elderly residents. This was reflected in only 25.3% of the sample studied were adherent to their medications. Health teaching for residents regarding medication regimens is crucial. Frances 2016^12^ revealed that health care providers’ education influences effective drug use in the elderly, this finding strongly agree with the current study.

Moreover, Frankfield et. al 2010^13^ and Shafik 2000^14^ reported that lower income and cost of drugs were associated with medication non-adherence this is an essential concern among older adults, with no significant relation in the current study. Also noticed a significant correlation between level of education of residents and their medication adherence among the study sample (P=0.05). On the other hand, there were no correlation between Age, gender, monthly income, chronic disorders, side effects, hospitalization, and medication adherence among study sample.

For inmates with insufficient information concerning the disease, the therapy and the expected outcomes also were included that group who did not take proper medication. In addition, the elderly residents suffered from several medications adverse events which have led to hospitalization in some cases. Regarding medication disposal, improper behaviors have been reported by our residents which negatively affects our environment as well as may lead to in appropriate behaviors as self-medication that may lead to severe outcomes including antibiotic resistance.^15^-^17^

The national medical insurance system was successfully covering thirteen residents in terms of physicians’ visits and follow ups and residents in terms of providing free medications. The medications provided followed the current guidelines of therapy for the required diseases.^18^ There is a real need for a gerontological nurse that educate residents on medication administration and to follow up on the compliance and adherence.

### Limitation of the study

It includes small sample size due to the availability of only two elderly homes in the study setting.

## CONCLUSION

The residents had chronic diseases out of 82.5% of with only 20.5% were having proper medical care exposure and education. This was reflected in their low medication adherence, adverse side effects and hospitalization. To overcome that and have better medical care exposure, extension of the national medical insurance system to include larger number of elderly population is suggested. It is also suggested to have a daily resident gerontological nurse and a weekly or biweekly visiting clinical pharmacist for such homes. Those can be assigned from the closest governmental hospital that belongs to the national medical insurance system to decrease the economic burden on such residences.
